# Cardiac specific transcription factor Csx/Nkx2.5 regulates transient-outward K^+^ channel expression in pluripotent P19 cell-derived cardiomyocytes

**DOI:** 10.1186/s12576-020-00748-z

**Published:** 2020-03-25

**Authors:** Tomoko Uchino, Ming-Qi Zheng, Yan Wang, Katsushige Ono

**Affiliations:** 1 Department of Pathophysiology, Oita University School of Medicine, Oita, Japan; 2 Department of Anesthesiology, Oita University School of Medicine, Oita, Japan; 3 Department of Cardiovascular Medicine, Hebei Medical University, Shijiazhuang, Hebei China

**Keywords:** Potassium channel, Csx/Nkx2.5, Cardiomyocytes, Transient outward current, Cardiogenesis, Pluripotency, P19CL6, Homeobox

## Abstract

The homeobox-containing gene Csx/Nkx2.5 codes several cardiac transcription factors and plays a critical role in early cardiogenesis. We investigated the effect of Csx/Nkx2.5 on the expression of cardiac ion channels using P19-derived cardiomyocytes. P19CL6 cells and P19CL6 cells with Csx/Nkx2.5 overexpression (P19CL6-Csx cells) were induced to differentiate into cardiomyocytes by treatment with dimethyl sulfoxide. Action potentials and membrane currents were measured by whole cell patch clamp at different differentiation stage: the early stage (1–5 days after beating had begun) and the late stage (10–15 days after beating). Expression of Csx/Nkx2.5 mRNA was increased as the differentiation stages advanced in both P19CL6 and P19CL6-Csx cells. In action potential configuration, maximal diastolic potentials in P19CL6-Csx cells exhibited more hyperpolarized potential (‒ 64.2 mV) than those in P19CL6 cells (‒ 54.8 mV, *p* < 0.01) in the early stage. In P19CL6 cells, among 6 different voltage-gated and ligand-operated K^+^ channels expressed during the early stage, the transient-outward K^+^ channel was most predominant. By overexpression of Csx/Nkx2.5, developmental decrease in the transient-outward K^+^ channel was suppressed. Homeobox-containing gene Csx/Nkx2.5 modifies the amount of distinct ionic channels, during differentiation periods, predominantly changing the expression of the transient-outward K^+^ channel.

## Introduction

A homeobox-containing gene Csx/Nkx2.5 is one of the cardiac-enriched transcription factors found by Komuro and Izumo [1]. Targeted disruption of murine Csx/Nkx2.5 results in embryonic lethality due to abnormal looping morphogenesis of the primary heart tube [2]. Recently, many different human Csx/Nkx2.5 mutations have been reported in patients with cardiac malformation such as atrial septal defects, atrioventricular conduction delays, ventricular septal defects, tetralogy of Fallot, and tricuspid valve abnormalities [3, 4]. These reports suggest that the main role of Csx/Nkx2.5 includes regulation of cardiac morphological differentiation. Moreover, its ability to protect the heart from stress has also been reported [5], suggesting that Csx/Nkx2.5 may have various effects on differentiation of the heart.

Establishment of an in vitro cardiomyocyte differentiation system has allowed us to study the function of ion channels in very early stages of differentiation. P19 embryonal carcinoma cells are a pluripotent cell line which can differentiate into cardiomyocytes after chemical induction by dimethyl sulfoxide (DMSO) [6–8]. P19CL6 cells were isolated from P19 cells by a limiting dilution method; P19CL6 cells can differentiate into cardiomyocytes more efficiently compared to P19 cells [9]. Furthermore, P19CL6 cells with Csx/Nkx2.5 overexpression (P19CL6-Csx cells) were reported to start spontaneously beating earlier and to differentiate more effectively than P19CL6 cells [10].

It is widely recognized that important electrophysiological changes occur during the embryonic development of mammalian hearts. The levels of expression and the biophysical and pharmacological properties of ion channels change during the course of development [11]. However, the mechanisms related to the development of the expression of ionic channels and their regulation by cardiac specific transcription factors are poorly understood. We hypothesized that Csx/Nkx2.5 has distinct effects on the differentiation/development of cardiac ion channels. Therefore, we investigated the effect of Csx/Nkx2.5 overexpression on the functional expression of cardiac ion channels using P19CL6 cells and P19CL6 cells transfected to overexpress Csx/Nkx2.5.

## Materials and methods

### Cell culture and differentiation

P19CL6 cells were cultured as described previously [9]. Briefly, P19CL6 cells were grown in 100 mm tissue culture dishes under adherent conditions with α-minimal essential medium (Invitrogen, Carlsbad, CA, USA) supplemented with 10% fetal calf serum (Hyclone, South Logan, UT, USA), penicillin (100 U mL^−1^), and streptomycin (100 µg mL^−1^) (growth medium), and were maintained in a 5% CO_2_ atmosphere at 37 °C. To induce differentiation under adherent conditions, P19CL6 cells were plated in a 60 mm tissue culture dish at a density of 3.7 × 10^5^ cells with growth medium containing 1% DMSO (differentiation medium). The medium was changed every other day. Days of differentiation were numbered consecutively after the 1st day of the DMSO application, day 0. Ten days after treatment with DMSO, most cells started beating spontaneously. We discriminated these cells based on their differentiation stage: early stage (10–15 days) and late stage (20–25 days), and experiments were performed on beating cardiomyocyte-like cells at these two stages.

### Reverse transcription (RT)-PCR

Total RNA was isolated from P19CL6 and P19CL6-Csx cells using an Isogen RNA extraction kit (Nippon Gene, Toyama, Japan), and RT-PCR was performed as described previously [5]. The primer sequences for Csx/Nkx2.5 were designed: 5′-TCT CCG ATC CAT CCC ACT TTA TTG-3′ for sense and 5′-TTG CGT TAC GCA CTC ACT TTA ATG-3′ for antisense. Amplification of α-actin mRNA was used an internal control for RT-PCR analysis. PCR conditions were 94 °C for 3 min, followed by 30 cycles 94 °C for 30 s, 56 °C for 30 s, and 72 °C for 1 min. PCR products were electrophoresed on 2% agarose gels and visualized by ethidium bromide staining. The densitometry of the bands was assessed via the NIH image 1.63 (National Institutes of Health, Springfield, VA, USA). Semi-quantitative evaluation of mRNA was performed by the ratio of Csx/Nkx2.5 mRNA densitometry to that of α-actin; the relative expression ratio of Csx/Nkx2.5 mRNA over α-actin mRNA in the early stage of P19CL6 cell was taken to be 1.0.

### Stable transformants

Establishment of P19CL6 cell lines stably overexpressing Csx/Nkx2.5 was done as described previously [10]. In brief, pcDNA3.1 plasmids containing cDNA of human wild-type Csx/Nkx2.5 were transfected into undifferentiated P19CL6 cells by the lipofection method with Tfx Reagents (Promega, Madison, WI, USA). Stable transformants were selected with 800 µg of neomycin (G418) per mL. Throughout the experiments, P19CL6 cells transfected with pcDNA3.1 plasmids containing vacant cDNA were used as parental P19CL4 cells for comparison.

### Preparation of single beating cells

Single cardiomyocyte-like cells were prepared by modifications of the methods described by Isenberg and Klockner [12]. Briefly, beating myocytes were mechanically isolated with a sterilized microscalpel and washed in a low Ca^2+^-medium. Subsequently, tissue fragments were incubated in enzyme-containing medium for 10–20 min at 37 °C. The dissociation of the tissue was completed in KB medium by gentle shaking at room temperature for 30 min. The isolated cells were plated on a 35-mm culture dish in differentiation medium and incubated for 12–24 h before doing experiments. Only spontaneous beating cells were used to measure ionic currents and action potentials.

### Electrophysiological recordings

For electrophysiological recordings, we used a whole cell patch-clamp technique throughout the study as described before [13]. Voltage clamp mode was used to measure ionic currents, and current clamp mode was used to measure action potentials using an EPC-8 (HEKA Elektronik, Lambrecht, Germany). The temperature of the external solution was kept at 37 °C with a chamber heating system (Bipolar Temperature Controller, model TC-202A, Harvard Apparatus, Holliston, MA, USA). Patch pipettes (2 to 3 MΩ electrical resistance filled with pipette solutions described below) were pulled from micro-glass capillaries (Drummond, Broomall, PA, USA) with Micropipette Puller, Model P-97 (Sutter Instrument, Novato, CA, USA). Series resistance was compensated electronically as much as possible without oscillation (60 to 75%). Capacitive artifacts were minimized by using the built-in circuitry of the amplifier. The remaining transients and linear leakage currents were eliminated by using p/4 subtraction (Pulse/Pulsefit, HEKA Elektronik). The amplifier output was cut-off filtered at 5 kHz, digitally sampled at 10 kHz by using an ITC-16 interface (Instrutech Corp., Great Neck, NY, USA), and stored on a computer under the control of a data acquisition program (Pulse/Pulsefit, HEKA Elektronik). For continuous action potential recording, the amplifier output was sampled with Power Lab (AD Instruments, Sydney, Australia) and stored on a computer with Chart software (AD Instruments, Sydney, Australia).

### Solutions

We used the following solutions to prepare single myocytes: (1) low Ca^2+^-medium: (in mmol L^−1^) 120 NaCl, 5.4 KCl, 5 MgSO_4_, 5 Sodium Pyruvate, 20 glucose, 20 taurine, 10 HEPES, with the pH adjusted to 6.9 with NaOH. (2) Enzyme medium: low Ca^2+^-medium supplemented with 1 mg mL^−1^ collagenase (type 2, Yakult, Tokyo, Japan) and 30 µmol L^−1^ CaCl_2_; 3) KB medium (mmol L^−1^): 85 KCl, 30 K_2_HPO_4_, 5 MgSO_4_, 1 EGTA, 2 Na_2_ATP, 5 Sodium Pyruvate, 5 creatine, 20 taurine, 20 glucose, pH adjusted to 7.2 with KOH.

For electrophysiological recordings, the following solutions were used. The internal solution used for the recording of transient-outward K^+^ current (*I*_to_), rapidly activating delayed rectifier K^+^ current (*I*_Kr_), slowly activating delayed rectifier K^+^ current (*I*_Ks_), and inwardly rectifier K^+^ current (*I*_K1_) was composed of (mmol L^−1^): 140 KCl, 1 MgCl_2_, 10 EGTA, 10 HEPES, 5 MgATP, pH adjusted to 7.2 with KOH. For recording ATP-activated K^+^ current (*I*_K,ATP_), EGTA and MgATP were reduced to 1 mmol L^−1^ and 0.1 mmol L^−1^, respectively, and 1 mmol L^−1^ Na_2_GDP and 5 mmol L^−1^ creatine phosphate were added to the internal solution. For recording acetylcholine-activated K^+^ current (*I*_K,ACh_), EGTA and MgCl_2_ were reduced to 1 mmol L^−1^ and 0.5 mmol L^−1^, respectively. MgATP was replaced with 2 mmol L^−1^ Na_2_ATP, and 0.2 mmol L^−1^ Na_2_GTP was added to the internal solution. For action potential recording, EGTA was reduced to 0.05 mmol L^−1^. The bath solution for recording action potentials and *I*_K,ATP_ consisted of the following millimolar concentrations: 140 NaCl, 5.4 KCl, 1.8 CaCl_2_, 1 MgCl_2_, 10 HEPES, 10 glucose, pH adjusted to 7.4 with NaOH. For *I*_to_ recording, 0.03 mmol L^−1^ TTX and 0.3 mmol L^−1^ CdCl_2_ were added to the above solution to eliminate Na^+^ current and Ca^2+^ currents, respectively. For the recording of other K^+^ currents, 0.3 mmol L^−1^ CdCl_2_ was added to the bath solution.

### Sources of test substances

TTX was purchased from Sankyo Co. Ltd (Tokyo, Japan). CdCl_2_ and 4-aminopyridine (4-AP) were purchased from Wako Pure Chemical Industries Ltd (Tokyo, Japan). All other chemicals were purchased from Sigma Co. (St. Louis. MO, USA). E-4031 was a gift from Eisai Pharmaceutical Co. (Tokyo, Japan), and chromanol 293B was a gift from Hoechst Marion Roussel (Frankfurt, Germany).

### Data analysis

All values were expressed as mean ± standard deviation (SD). Two-way ANOVA followed by a Bonferroni post hoc test was used for multiple comparisons, and statistical significance was considered when *p* values were less than 0.05.

## Results

### Expression of Csx/Nkx2.5 mRNA during differentiation in P19CL6 cells and P19CL6-Csx cells

**Fig. 1:**
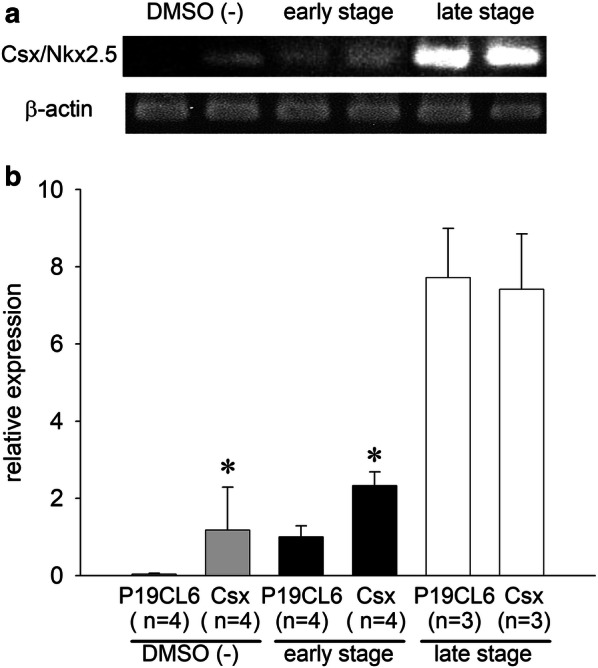
The expression of Csx/Nkx2.5 mRNA analyzed by semi-quantitative RT-PCR. **a** Representative ethidium bromide staining of PCR products of P19CL6 cells (lanes 1, 3, 5) and P19CL6-Csx cells (lanes 2, 4, 6) during differentiation period. **b** Relative expression of Csx/Nkx2.5 mRNA was shown after normalizing to the expression of β-actin in each lane

To confirm the difference in expression of Csx/Nkx2.5 mRNA in P19CL6 cells vs. P19CL6-Csx cells, we performed an RT-PCR assay at different differentiation stages. Figure 1 shows the developmental increase in Csx/Nkx2.5 mRNA expression in P19CL6 cells. In P19CL6-Csx cells, Csx/Nkx2.5 mRNA overexpression was recognized prior to the treatment with DMSO. In the early stage, Csx/Nkx2.5 mRNA expression in P19CL6-Csx cells was 2.3 times greater than that in P19CL6 cells. According to the cell differentiation, the Csx/Nkx2.5 mRNA expression in the late stage was markedly upregulated in both cell groups, ultimately reaching the same expression level regardless of the Csx/Nkx2.5 overexpression.

### Action potential configurations of P19CL6 cell-derived and P19CL6-Csx cell-derived cardiomyocytes

**Fig. 2:**
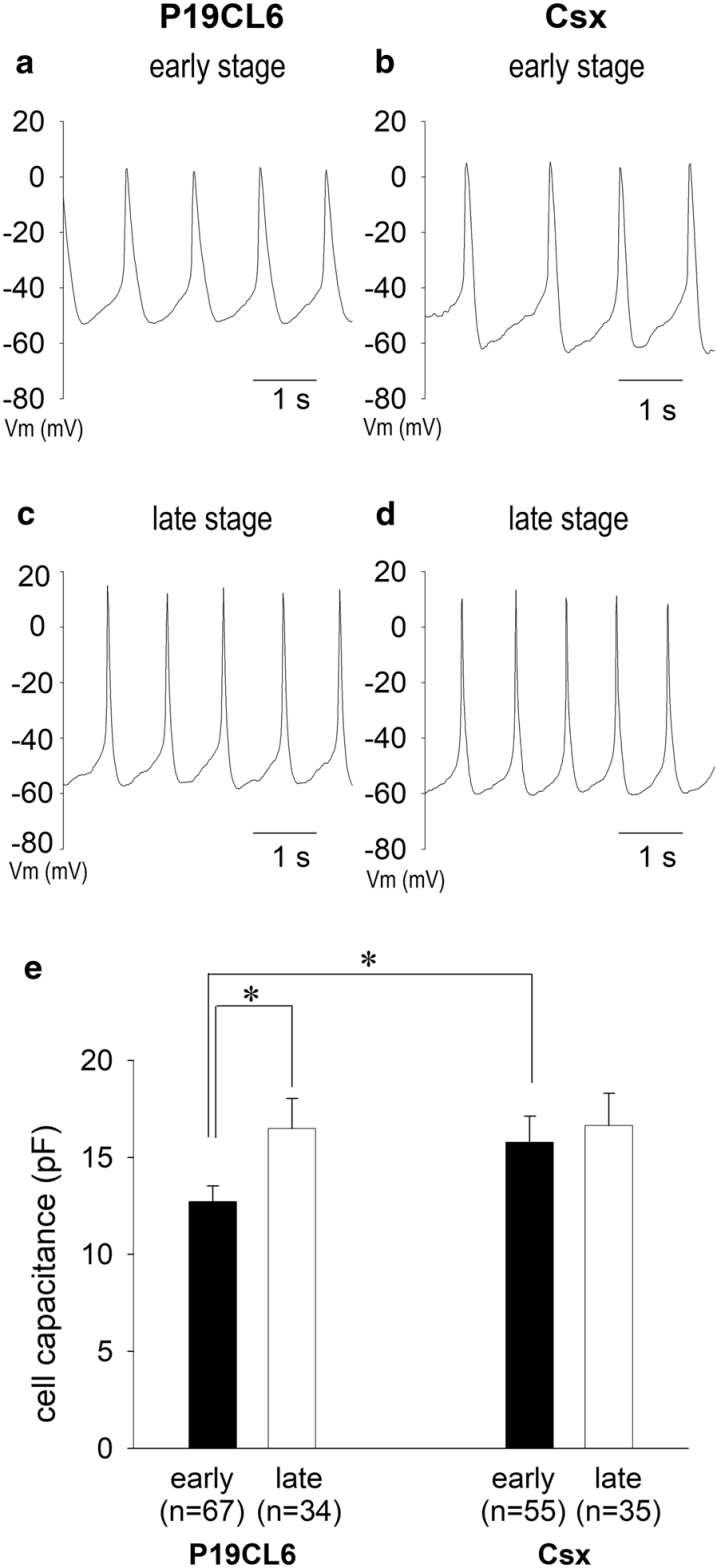
Change in action potentials and cell size during differentiation. Typical action potential recordings of P19CL6-derived cardiomyocytes (**a**, **c**) and P19CL6-Csx-derived cardiomyocytes (**b**, **d**). Developmental change in cell capacitance of P19CL6-derived cardiomyocytes and P19CL6-Csx-derived cardiomyocytes (**e**). Action potentials were recorded in current clamp mode in the early stage (**a**, **b**) and in the late stage (**c**, **d**) of differentiation. Each bar indicates the mean of total membrane capacitance values in the early stage (filled bars) and the late stage (open bars) in panel (**e**). *p < 0.05 compared with P19CL6 in the early stage

We examined action potential configurations of cardiomyocyte-like cells derived from P19CL6 cells and P19CL6-Csx cells. Figure 2a–d shows representative action potentials in a P19CL6 cell and a P19CL6-Csx cell. Action potential configurations of spontaneous beating cells resembled those in pacemaker cells such as sinoatrial (SA) node cells in terms of slow diastolic potentials and relatively narrow action potentials. Parameters of action potential configurations are shown in Table 1. Beating rate showed no significant change in both cell lines. In P19CL6-Csx cells, maximal diastolic potentials (MDP) were more hyperpolarized than those of P19CL6 in each developmental stage. Action potential duration to 50% repolarization (APD_50_) was shortened as differentiation stage advanced in both cell lines.

**Table 1:** Action potential parameters of P19CL6-derived cardiomyocytes and P19CL6-Csx-derived cardiomyocytes

	P19CL6		P19CL6-Csx	
	Early stage (*n* = 17)	Late stage (*n* = 11)	Early stage (*n* = 17)	Late stage (*n* = 8)
Beating rate (bpm)	65.9 ± 21.2	73.3 ± 47.1	72.5 ± 29.2	80.6 ± 24.6
Maximal diastolic potential (mV)	‒ 54.8 ± 5.4	‒ 56.7 ± 8.2	‒ 64.2 ± 7.2*	‒ 59.4 ± 4.7
Maximal upstroke velocity (V s^−1^)	3.2 ± 1.4	3.8 ± 1.9	3.3 ± 1.6	4.1 ± 1.6
APD_50_ (ms)	77.4 ± 19.7	53.8 ± 28.1*	76.6 ± 27.5	48.4 ± 20.0^†^

Each value was collected as an average of randomly assigned 10 consecutive action potentials from cells indicated in parentheses in number, and are shown as mean ± SD

*APD*_*50*_ action potential durations measured at 50% repolarization

* *p* < 0.05 compared with P19CL6 cells in the early stage

^†^*p* < 0.05 compared with P19CL6-Csx cells in the early stage

### Developmental changes in cell size

Developmental changes in cell size are summarized in Fig. 2e by monitoring their cell capacitances. In P19CL6 cells, cell capacitance was increased significantly as development progressed (12.7 ± 0.8 pF to 16.5 ± 1.5 pF). However, no appreciable change in cell capacitance (15.8 ± 1.3 pF to 16.6 ± 1.7 pF) was detected in P19CL6-Csx cells. Cell capacitance of P19CL6-Csx cells in the early stage was significantly larger than that of P19CL6 cells, indicating that P19CL6-Csx cells were matured in size by the time of early stage differentiation.

### Developmental changes in K^+^ channels

It has been known that K^+^ channels are expressed in the heart from the early embryonic period, and they are modified throughout the differentiation period. In cardiomyocytes, K^+^ currents could be roughly sorted into three groups: transient outward currents (*I*_to1_, *I*_to2_), voltage-dependent rectifier K^+^ currents (*I*_Kr_, *I*_Ks_, *I*_K1_) and ligand-operated K^+^ currents (*I*_K, ATP_, *I*_K,ACh_). To clarify the electrical contribution of these subpopulation of K^+^ currents, known selective blockers, such as 5 mmol L^−1^ 4-AP for *I*_to_, 5 µmol L^−1^ E4031 for *I*_Kr_, and 50 µmol L^−1^ chromanol 293B for *I*_Ks_, were used.

### Transient outward current

**Fig. 3:**
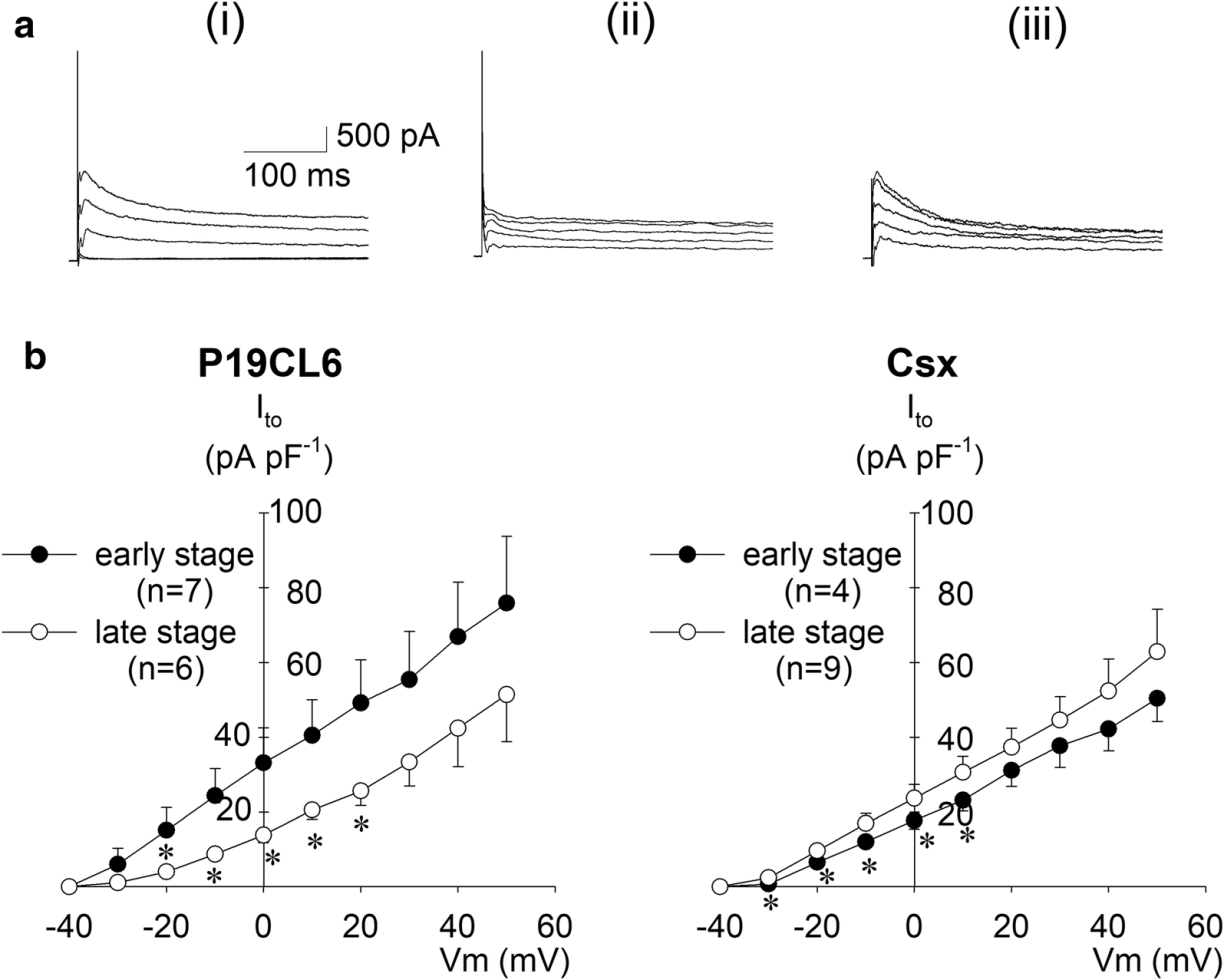
Transient outward currents (*I*_to_) in P19CL6-derived cardiomyocytes and P19CL6-Csx-derived cardiomyocytes. **a** Representative current traces from P19CL6-derived cardiomyocytes in the early stage without 4-AP (i), with 5 mmol L^−1^ 4-AP (ii), and (iii) the difference between the two (i)–(ii). Currents were elicited by depolarizing pulses from a holding potential of ‒ 80 mV to the test potentials between ‒ 30 and + 50 mV (20 mV increments). **b** Current densities were plotted against test potentials in P19CL6-derived cardiomyocytes (left panel) and P19CL6-Csx-derived cardiomyocytes (right panel). *p < 0.05 compared with P19CL6 cells in the early stage

Transient outward currents are strictly composed of two constituents: 4-AP sensitive K^+^ currents (*I*_to1_) and Ca^2+^-activated Cl^−^ currents (*I*_to2_). *I*_to_ was nevertheless defined as the 4-AP sensitive current in this study. Current amplitude was measured by subtraction of the outward peak current from the steady-state current level at the end of the test pulse, and calculated as the difference in amplitude before and after 4-AP application. Figure 3a shows typical current traces of P19CL6 cells in the early stage before (i) and after (ii) application of 5 mmol L^−1^ 4-AP, and the subtracted current (iii) the difference, (i)–(ii), representing *I*_to_. Figure 3b shows current–voltage relationships of *I*_to_ in P19CL6 cells and P19CL6-Csx cells. In P19CL6 cells, current density of *I*_to_ in the early stage was significantly larger than that of the late stage, while no significant difference of *I*_to_ current density between early and late stages was observed in P19CL6-Csx cells.

### Voltage-dependent rectifier K^+^ currents; *I*_Kr_, *I*_Ks_, and *I*_K1_

**Fig. 4:**
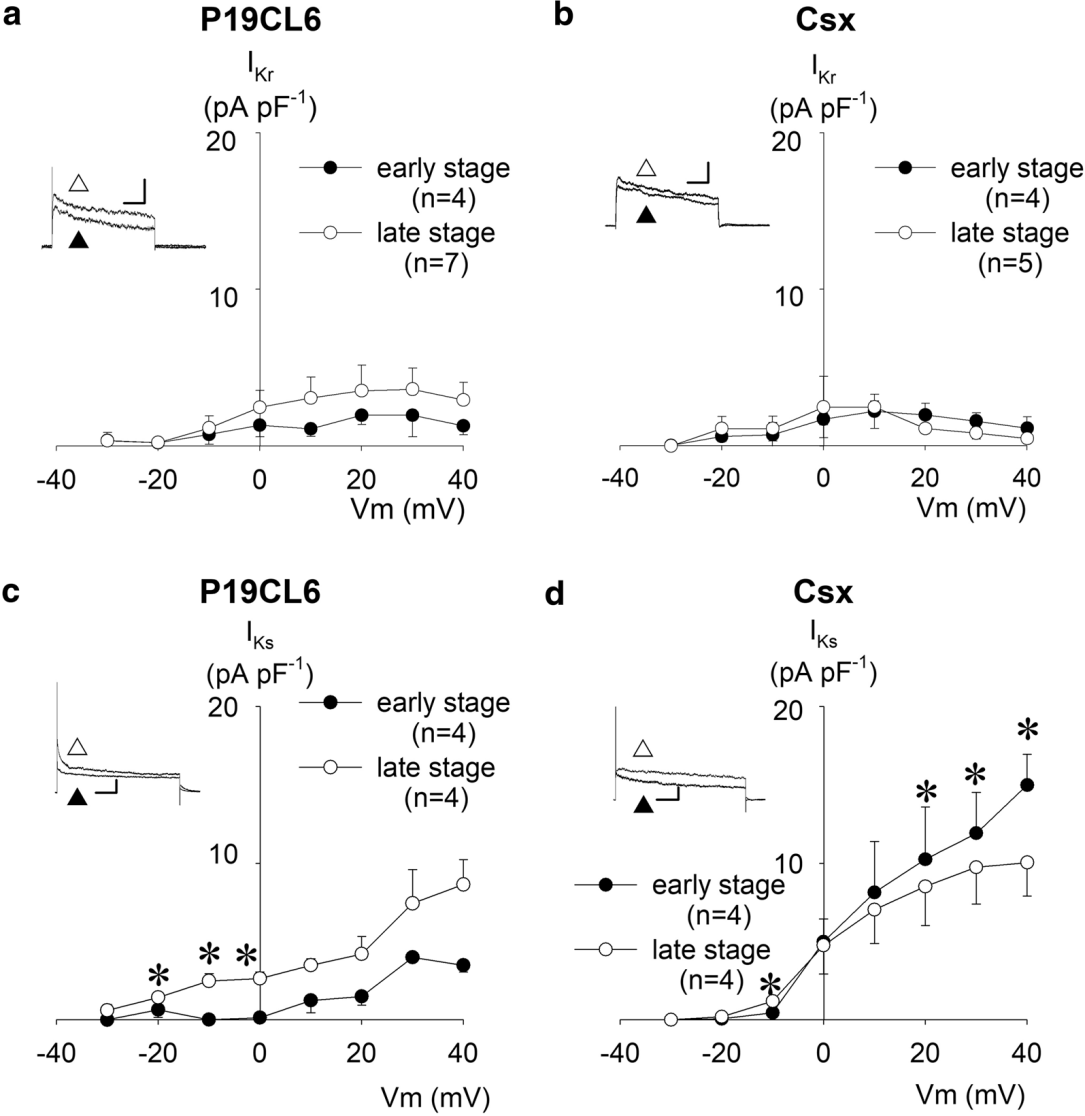
Delayed rectifier K^+^ currents expressed in P19CL6-derived cardiomyocytes and P19CL6-Csx-derived cardiomyocytes. Current–voltage relationships of E4031-sensitive currents (*I*_Kr_) (**a**, **b**), and chromanol 293B-sensitive currents (*I*_Ks_) (**c**, **d**). Each inset shows representative current traces (at 0 mV in *I*_Kr_, at + 30 mV in *I*_Ks_,) before (white up-pointing triangle) and after (black up-pointing triangle) application of each inhibitor. Scales of insets are 200 ms and 100 pA for *I*_Kr_, 500 ms and 200 pA for *I*_Ks_. *I*_Kr_ was elicited by 1 s-depolarizing pulses from a holding potential of ‒ 40 mV to the test potentials between ‒ 30 and + 40 mV (10 mV increments). *I*_Ks_ was elicited by the same protocol as *I*_Kr_, except for the duration of depolarizing pulse (3 s). Current amplitude was measured at the end of the depolarizing test pulse and normalized by cell capacitance. *p < 0.05 compared with P19CL6 cells in the early stage

We evaluated two components of delayed rectifier currents, *I*_Kr_ and *I*_Ks_. These ionic channels were previously reported as being present in neonatal mouse cardiomyocytes [14]. A selective *I*_Kr_ blocker, 5 µmol L^−1^ E4031, and a selective *I*_Ks_ blocker, 50 µmol L^−1^ chromanol 293B, were used to discriminate between these two components. As shown in Fig. 4a, b, *I*_Kr_ amplitude was very small in contrast to that of *I*_to_ in these cell lines. In this experimental condition without 4-AP, *I*_Kr_ and *I*_to_ coexisted in a trace because their activation time and voltage overlapped, therefore, a fairly small *I*_Kr_ was hidden behind the large *I*_to_ in the absence of 4-AP. *I*_Kr_ was substantially unchanged throughout the early and late differentiation stages, both in P19CL6 and P19CL6-Csx cells. The *I*_Kr_ showed inward-going rectification at + 20 mV (P19CL6 cells) and + 10 mV (P19CL6-Csx cells) or more depolarized potentials, similarly to the *I*_Kr_ in native cardiac myocytes.

To determine the amount of current contribution by *I*_Ks_ to the total outward current, a specific *I*_Ks_ blocker was applied to the bath solution after observation of control current. *I*_Ks_ was defined as 50 µmol L^−1^ chromanol 293B sensitive current in this study. As shown in Fig. 4c, d, possible contamination of *I*_to_ was detected, since a large current was revealed at the first component after the depolarization in comparison with delayed rectifiers. *I*_Ks_ was expressed in both cell lines, and a significant difference between these cell lines was observed in the early stage. It is worthily stressed that *I*_Ks_ in P19CL6 cells in the early stage was markedly small in comparison with those in the late stage up to the membrane potentials of + 40 mV: 0.1 ± 0.6 pA pF^−1^ (early stage) vs 2.5 ± 0.4 pA pF^−1^ (late stage) at 0 mV (*p* = 0.02), for instance, consequently resulting in a prolongation of APDs in this stage (Table 1).

**Fig. 5:**
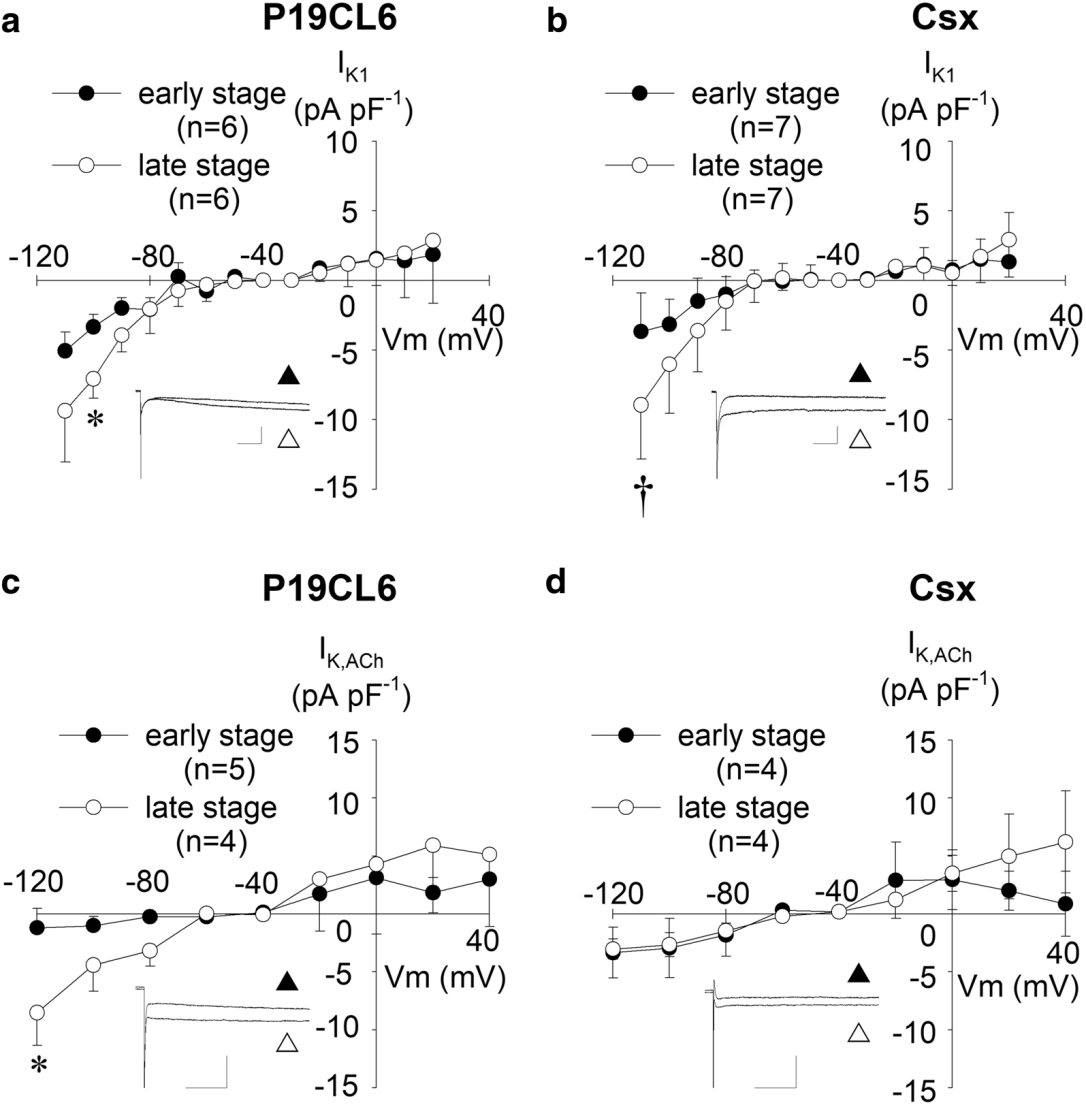
Inward rectifier K^+^ currents (*I*_K1_) and ACh-activating K^+^ currents (*I*_K,ACh_) in P19CL6-derived cardiomyocytes and P19CL6-Csx-derived cardiomyocytes. Current–voltage relationships of *I*_K1_ (**a**, **b**) and *I*_K,ACh_ (**c**, **d**). Scales of insets: 50 ms and 200 pA for *I*_K1_, 50 ms and 500 pA for *I*_K,ACh_. *I*_K1_ currents were elicited by hyperpolarizing and depolarizing pulses from a holding potential of ‒ 40 mV to the test potentials between ‒ 110 and + 20 mV (10 mV increments). Each inset shows representative current traces at ‒ 110 mV before (white up-pointing triangle) and after (black up-pointing triangle) application of 200 µmol L^−1^ Ba^2+^. *I*_K,ACh_ was elicited by hyperpolarizing and depolarizing pulses from a holding potential of ‒ 50 mV to the test potentials between ‒ 120 and + 40 mV (20 mV increments). Each inset shows representative current traces at ‒ 120 mV before (white up-pointing triangle) and after (black up-pointing triangle) application of 20 µmol L^−1^ carbachol. *p < 0.05 compared with P19CL6 cells in the early stage, and ^†^*p* < 0.05 compared with P19CL6-Csx cells in the early stage

*I*_K1_ is widely known to be sensitive to Ba^2+^ ions [15]. Therefore we evaluated *I*_K1_ as the difference in current with or without application of 200 µmol L^−1^ Ba^2+^. As depicted in Fig. 5a, b, developmental increase in *I*_K1_ was identified in hyperpolarized potentials (− 110 mV and − 100 mV) in both cell lines. These Ba^2+^-sensitive currents exhibited pronounced inward rectification, and only a very small current was observed at depolarized potentials of − 60 mV or more.

### Ligand-operated K^+^ currents; *I*_K,ACh_ and *I*_K,ATP_

**Fig. 6:**
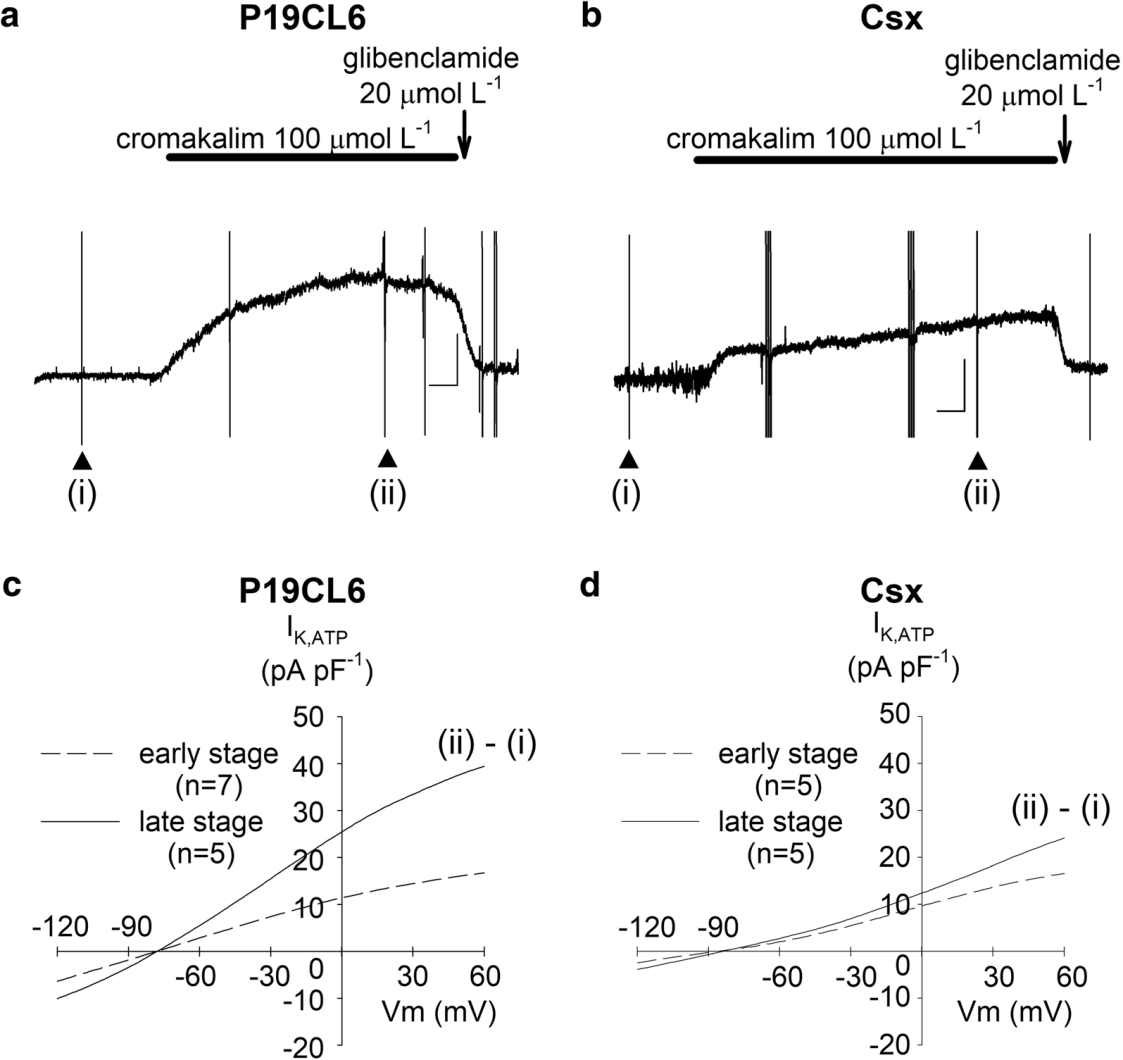
ATP-sensitive K^+^ currents (*I*_K,ATP_) in P19CL6-derived cardiomyocytes and P19CL6-Csx-derived cardiomyocytes. Sample current recordings at a holding potential of ‒ 40 mV (**a**, **b**). Instantaneous current–voltage relationships (**c**, **d**) were obtained by ramp pulses from + 60 mV to ‒ 120 mV at the rate of ‒ 0.18 V/s, before (i) and after (ii) application of 100 µmol L^−1^ cromakalim. Group data (*n* = 4) for the cromakalim-sensitive current, *I*_K,ATP_ (ii)–(i) are plotted in the early stage (dotted line) and in the late stage (solid line)

*I*_K,ACh_ was defined as the current component activated by 20 µmol L^−1^ carbachol in this study. As shown in Fig. 5c, d, *I*_K,ACh_ was detected in both cell lines. However, no significant developmental change and no significant difference between P19CL6 cells and P19CL6-Csx cells were observed except the current at the potentials of − 120 mV. *I*_K,ATP_ was defined as the current activated by 100 µmol L^−1^ cromakalim. Figure 6a shows continuous recording of the outward current at a potential of − 40 mV with or without the presence of an *I*_K,ATP_ opener (cromakalim) or an *I*_K,ATP_ blocker (glibenclamide). To obtain current–voltage relationships of *I*_K,ATP_, a ramp pulse (+ 60 mV to − 120 mV, − 0.18 V s^−1^) was applied at the points indicated (Fig. 6a, b). The initial ramp pulse trace was obtained prior to the application of cromakalim (i), and a second trace when eliciting outward current, immediately after cromakalim perfusion (ii). Current–voltage relationships of subtracted currents (ii)–(i) are shown in Fig. 6c, d. Opening of *I*_K,ATP_ was observed in both cell lines; a large density in *I*_K,ATP_ was observed in P19CL6 cells in the late stage.

## Discussion

The present study demonstrates developmental changes of K^+^ currents in P19CL6 cells, and their regulation by overexpression of homeobox-containing transcription factor, Csx/Nkx2.5. Although Csx/Nkx2.5 has recently been identified as a key transcription factor for the Ca_V_3.2-T-type Ca^2+^ channel expression [16], a distinct role of Csx/Nkx2.5 on the expression of the transient-outward K^+^ channel was elucidated for the first time in P19CL6 cell-derived cardiomyocytes.

The electrical and mechanical mechanisms governing the precise and highly organized actions responsible for electrical propagation in the heart are extremely complex, requiring coordinated neural and humoral factors in the healthy and pathological conditions [17–24]. Such regulatory coordination also depends on the developmental changes in the cardiac substrates that are responsible for actions of transcriptional/translational feedback modifications. In recent years, in vitro models using iPSC-derived cell types or pluripotent cell lines have rapidly emerged as a powerful genetic system to study cardiac development and function [25, 26].

The P19CL6 cell line is a clonal derivative isolated from murine P19 embryonal carcinoma cells by the limiting dilution methods [9]. Unlike P19 cells which show low efficacy of differentiation into cardiomyocytes, P19CL6 cells efficiently differentiate (more than 80%) into beating cardiomyocytes with adherent conditions when treated with 1% DMSO, thus mimicking the events of early cardioembryogenesis [5, 9, 10].

In P19CL6 cells, most cardiac K^+^ channels, *I*_to_, *I*_Kr_, *I*_Ks_, *I*_K1_, *I*_K,ACh_, and *I*_K,ATP_, were expressed at the early stage. The expression of these channels has been recognized in mouse embryo [27]. In P19CL6 cells, *I*_to_ was a dominant component of all K^+^ currents throughout the entire differentiation period as reported for mouse ES cell-derived cardiomyocytes [28]. *I*_to_ is an important repolarizing current on action potentials, especially in the early differentiation period in cardiogenic mesodermal cells. Nevertheless, the APD_50_ in P19CL6 cells was shortened as the differentiation stage advanced, despite the decreasing density of *I*_to_. In mouse ES cell-derived cardiomyocytes, APD_50_ was not altered by 4-AP application in the late differentiation stage, and was only prolonged by 11% by 4-AP application in the early differentiation stage [29]. In 1-day-old neonatal mouse ventricular myocytes, APD was not altered by 4-AP [30]. Taken together, the contribution of *I*_to_ to the repolarization phase of action potentials may be small in late embryonal or neonatal cardiomyocytes. It is postulated that *I*_to_ density in myocytes has a negative correlation with cardiac hypertrophy [31–34]. In zebrafish, overexpression of Csx/Nkx2.5 was shown to enlarge the heart [35]. In our study, the developmental increase in cell capacitance was negatively correlated with *I*_to_ density, suggesting the possible contribution of Csx/Nkx2.5 for cardiac hypertrophy in mammalian hearts.

In contrast to the large amplitude of *I*_to_, delayed rectifier K^+^ currents, *I*_Kr_ and *I*_Ks_, were observed to have a relatively small amplitude at the early stage in P19CL6 cells (Fig. 4). These currents were detected as being nearly identical in terms of current density in the early stage, and then *I*_Ks_ became dominant in the late stage. This developmental change of the dominant component in delayed rectifiers was consistent with that for mouse embryonal ventricular myocytes [15]. *I*_K1_, *I*_K,ACh_, and *I*_K,ATP_ were all recorded in P19CL6-derived myocytes in this study. It is widely accepted that these three current components contribute to the resting membrane potential (RMP). *I*_K,ATP_ develops progressively before birth in accordance with the establishment of RMP. Therefore, it is speculated that *I*_K,ATP_ is responsible for the determination of RMP in embryonic heart [36]. The opening of *I*_K,ACh_ was reported as a background current and contributed to diastolic depolarization in rabbit SA node cells [37]. In P19CL6-derived myocytes, the density of *I*_K,ATP_ was largest in these three current components at a membrane potential of ‒ 55 mV, namely, the approximate maximal diastolic potential in P19CL6 cells, suggesting that *I*_K,ATP_ is the most effective determinant of RMP in cardiogenic cells at the early developmental stage. Therefore, it is of great advantage to apply patch clamp methods to P19CL6-derived myocytes for studying distinct transcription factor-dependent transcriptional regulation of the ionic channels, especially in K^+^ channels.

A homeobox-containing transcription factor, Csx/Nkx2.5, was initially reported by Komuro and Izumo [1]. Various roles for Csx/Nkx2.5 in cardiac development were suggested later on [1–3, 10, 27, 31, 38]. We have previously shown the transcriptional action of Csx/Nkx2.5 on the voltage-dependent inward current channels; overexpression of Csx/Nkx2.5 upregulated Ca_V_3.2-T-type Ca^2+^ channel expression and had no effect on the L-type Ca^2+^ channel or the voltage-dependent Na^+^ channel [39]. Therefore, we focused the present study of Csx/Nkx2.5 actions on the expression of K^+^ channels in this study. In this context, we demonstrated for the first time that developmental changes in the distinct K^+^ channels, *I*_to_ and *I*_Ks_, are modified by Csx/Nkx2.5. Postnatal developmental change in *I*_to_ density in myocytes is correlated with shortening of the action potential duration in ventricular cells [15]. In the embryonic period of mice, *I*_to_-like currents were also reported to have three different types of inactivation kinetics: rapidly inactivating type, slowly inactivating type, and non-inactivating type [40]. Each current was expressed dependently in the chamber, and independently of embryonic age. In atrium, rapidly inactivating current was dominantly expressed, whereas slowly and non-inactivating current were dominantly expressed in ventricle. Ionic current by the slow component was estimated to be dominant than that by the rapidly inactivating one in ventricular myocyte [40]. Actually, in this study, the proportion of the slowly inactivating current to the total I_to_ was increased as differentiation advanced in P19CL6 cells, and was predominant from the early stage in P19CL6-Csx cells (data not shown). Taken together, it is suggested that P19CL6 cells gain their mature properties of *I*_to_ as the differentiation stage advances, and Csx/Nkx2.5 promotes its modification. Interestingly, Csx/Nkx2.5 mRNA expression in P19CL6 and P19CL6-Csx cells was similarly increased in the late differentiation stage (Fig. 1). It is therefore speculated that transcription factor Csx/Nkx2.5 affects one or more pathway that have inhibitory signals in the promotion region of some *I*_to_ channel genes only at the early differentiation stage, and furthermore, that the regulation mechanism is diminished toward the cardiac differentiation. Another cardiac specific transcription factor, GATA4, enhances the promoter of the K_V_4.2 (*I*_to_) K^+^ channel gene synergistically with Csx/Nkx2.5 [41], partially supporting our electrophysiological data. Recently, a minK-lacZ (minimal K^+^ channel, KCNE-1) mouse line has been utilized to track the development of the mouse conduction system, indicating that minK is a potential downstream target for Csx/Nkx2.5 [42]. Moreover, Csx/Nkx2.5-dependent minK gene dosage action was postulated in the crossed minK-lacZ knock-in mouse into the Csx/Nkx2.5 haplo-insufficiency mouse line [43]. Based on the findings in this study, augmentation of *I*_Ks_ in Csx/Nkx2.5 overexpression cells could be attributed to the expression of the β-subunit of *I*_Ks_ (minK) rather than to the pore-forming α-subunit (*K*_VLQT1_).

Even though our work presents a potential role of Csx/Nkx2.5 on the regulation of electrophysiological feature of developing cardiomyocytes, some important limitations need to be considered in further clarification of transcriptional modulation cardiac ion channels by the transcription factor. First, our study only considers ionic currents by use of patch clamp technique without identifying ion channel/isoform species. For this purpose, RT-PCR or other molecular biological evaluation are definitely needed. Also, it has been proposed that some ion channels may need distinct accessory proteins for the function maturation. Quantitative monitoring of gene expression patterns with a complementary DNA microarray would also be helpful. Second, the proposed function of Csx/Nkx2.5 may not place a role during steps in cardiogenesis, as P19CL6 cells are not finally confirmed to be differentiated into ventricular/atrial/sinus cardiomyocytes. Postulated roles of Csx/Nkx2.5 could be remediated with embryonic heart cells or iPS-derived heart cells. Another limitation of P19CL6 cells with Csx/Nkx2.5 overexpression in this study is the high expression system of this transcription factor in the cell. Further investigations are required to clarify the cellular signals that might exist between Csx/Nkx2.5 and these channels expression.

## Conclusions

We have investigated developmental changes in K^+^ currents in P19CL6 cell-derived cardiomyocytes, and found that the expression of *I*_to_ and, to a lesser extent, I_Ks_ was regulated by the cardiac specific homeobox-containing gene Csx/Nkx2.5. Our results suggest that the transcription factor Csx/Nkx2.5 promotes cell differentiation by means of regulating expression of distinct K^+^ channels.

## Data Availability

The datasets generated during and/or analyzed during the current study are available in the Oita University School of Medicine repository. They are also available from the corresponding author on reasonable request.

## Abbreviations

DMSO: Dimethyl sulfoxide
4-AP: 4-Aminopyridine
*I*_to_: Transient-outward K^+^ current
*I*_to1_: 4-AP sensitive transient-outward K^+^ currents
*I*_to2_: Ca^2^^+^-activated Cl^−^ currents
*I*_Kr_: Rapidly activating delayed rectifier K^+^ currents
*I*_Ks_: Slowly activating delayed rectifier K^+^ current
*I*_K1_: Inwardly rectifier K^+^ current
*I*_K,ATP_: ATP-activated K^+^ current
*I*_K,A__C__ch_: Acetylcholine-activated K^+^ current
MDP: Maximum diastolic potentials;
APD: Action potentials duration
APD_50_: The duration of 50% repolarization of action potentials
